# Local Community Assembly Mechanisms and the Size of Species Pool Jointly Explain the Beta Diversity of Soil Fungi

**DOI:** 10.1007/s00248-024-02374-3

**Published:** 2024-04-11

**Authors:** Hua Xing, Wuwei Chen, Yu Liu, James F. Cahill

**Affiliations:** 1 ECNU-Alberta Joint Lab for Biodiversity Study, Tiantong Forest Ecosystem National Observation and Research Station, School of Ecological and Environmental Sciences, East China Normal University, 500 Dongchuan Road, Minhuang District, 200241 Shanghai China; 2 Department of Biological Sciences, University of Alberta, Edmonton, AB T6G 2E9 Canada; 3 Qingyuan Bureau Natural Resources and Planning, Qingyuan, 323800 China; 4 Shanghai Institute of Pollution Control and Ecological Security, Shanghai, 200082 China

**Keywords:** Beta diversity, Community assembly processes, Functional groups of fungi, Species pool, Deterministic processes, Stochastic processes

## Abstract

Fungi play vital regulatory roles in terrestrial ecosystems. Local community assembly mechanisms, including deterministic and stochastic processes, as well as the size of regional species pools (gamma diversity), typically influence overall soil microbial community beta diversity patterns. However, there is limited evidence supporting their direct and indirect effects on beta diversity of different soil fungal functional groups in forest ecosystems. To address this gap, we collected 1606 soil samples from a 25-ha subtropical forest plot in southern China. Our goal was to determine the direct effects and indirect effects of regional species pools on the beta diversity of soil fungi, specifically arbuscular mycorrhizal (AM), ectomycorrhizal (EcM), plant-pathogenic, and saprotrophic fungi. We quantified the effects of soil properties, mycorrhizal tree abundances, and topographical factors on soil fungal diversity. The beta diversity of plant-pathogenic fungi was predominantly influenced by the size of the species pool. In contrast, the beta diversity of EcM fungi was primarily driven indirectly through community assembly processes. Neither of them had significant effects on the beta diversity of AM and saprotrophic fungi. Our results highlight that the direct and indirect effects of species pools on the beta diversity of soil functional groups of fungi can significantly differ even within a relatively small area. They also demonstrate the independent and combined effects of various factors in regulating the diversities of soil functional groups of fungi. Consequently, it is crucial to study the fungal community not only as a whole but also by considering different functional groups within the community.

**Supplementary Information:**

The online version contains supplementary material available at 10.1007/s00248-024-02374-3.

## Introduction

Fungi comprise a highly diverse group of organisms, estimated to include 2.2–3.8 million species [1]. They play crucial roles in regulating plant diversity and productivity in terrestrial ecosystems [2, 3]. Fungi participate in various nutritional relationships with plants, such as saprotrophic, mutualistic, and parasitic interactions [4]. These organisms are classified into different functional guilds based on their ecological roles and nutritional strategies (e.g., mycorrhizal fungi, pathogenic fungi, and saprotrophic fungi) [5]. Mycorrhizal fungi, mainly consisting of arbuscular mycorrhizal (AM) fungi and ectomycorrhizal (EcM) fungi, can benefit plants by providing nutrients and defending against pathogens [6–10]. Generally, EcM fungi have better access to resources in organic form than AM fungi, and EcM fungi have stronger antagonism against pathogenic fungi than AM fungi, thereby providing better defenses to their host plants [11–13]. Saprotrophic fungi, which decompose plant litter [14], play an important role in both carbon and nutrient turnover [15]. In addition, the presence of pathogenic fungi in natural ecosystems contributes to maintaining plant species diversity by impeding plant growth and reproduction, thereby preventing any single plant species from becoming predominant [16–19]. While factors influencing soil fungal diversity have been extensively studied in various ecosystems [20–22], there is still a significant knowledge gap in forest systems. One complexity lies in the fact that different fungal groups confer distinct functions, and it remains unclear to what extent each functional group is governed by shared community assembly processes or exhibits idiosyncratic behaviors (such as being influenced only by deterministic, stochastic processes, or the size of the local species pool).

The biodiversity and structure of soil functional fungal communities have received considerable attention [14, 23–25], and it is recognized that different factors may drive the structures of these communities. However, it is still an open question whether these different findings may arise from different studies or whether different fungal groups exhibit distinct responses even within a single community. For instance, when a functional group, such as AM or EcM fungi, is studied separately, it may be influenced by plant community composition and/or soil abiotic factors [26–31]. When considering the drivers of both functional groups together, results have been variable. Some studies have found that both EcM and saprotrophic fungi were influenced by plant communities and soil abiotic factors [32]. Other studies have shown that EcM fungi were primarily affected by plant communities, soil abiotic factors, and geographical distance, whereas saprotrophic fungi were only influenced by geographical distance [33]. Furthermore, it has been observed that mycorrhizal fungi were mainly determined by plant communities, whereas saprotrophic fungi were influenced by both plant communities and soil abiotic factors [34]. Regarding the three functional groups, some studies have suggested that plant communities explain the largest proportion of variation in plant-pathogenic, AM, and EcM fungi, while environmental and spatial factors account for smaller proportions [35]. Based on these findings, it is necessary to assess the presence of universal governing factors or idiosyncratic responses for different functional fungal communities in a single community.

Beta diversity, proposed by Whittaker in 1960, can not only reflect the diversity distance relationship between samples but also reflect the degree of differentiation between communities, which is related to the complexity of the environment [36]. For example, high beta diversity means that the community differentiation is high and the community structure is complex. Beta diversity plays a crucial role in shaping cross-scale biodiversity patterns [37], so it is urgent to study the beta diversity patterns of fungal communities. There are various ways to quantify beta diversity [38, 39], and it is influenced by both alpha and gamma diversities [40, 41]. The species pool, representing the complete set of species capable of naturally dispersing, establishing, and persisting in a specific area [42, 43], can have varying effects on community structure depending on the study scale [44]. Although previous studies have examined beta diversity patterns along latitudinal gradients [37, 45], there is limited research on beta diversity patterns of soil fungi in forest ecosystem plots based on different subplots. Investigating alpha, beta, and gamma diversity scales is applicable not only to the overall soil fungal community but also to its functional groups.

Community assembly processes can be categorized into deterministic and stochastic processes [46, 47]. Deterministic processes refer to the biological and environmental selection mechanisms that influence communities, while stochastic processes emphasize dispersal and ecological drift [48–50]. Previous studies have explored the mechanisms underlying community assembly of individual soil functional groups of fungi in specific ecosystems [51–53]. For example, Wang et al. studied EcM fungal communities of five common pine plant species in Inner Mongolia, China, and the results showed that both deterministic and stochastic processes jointly drove the community association of EcM fungi, but the stochastic process was dominant [52]. In addition, through the study of EcM fungal communities in young, intermediate, and old forests in subtropical ecosystems in China, Gao et al. found that EcM fungal communities are affected by environmental selection and dispersal limitation in old forests, but only by environmental selection in young, intermediate, and whole forests [53]. However, these studies have primarily focused on the assembly processes of individual functional groups of fungi and overlooked potential differences among other fungal guilds [54].

The size of the species pool and diverse community assembly mechanisms, such as heterogeneous environments and dispersal processes, may directly or indirectly influence the beta diversity of a community. For example, previous studies on plant communities have indicated that changes in beta diversity across broad biogeographic gradients are more likely driven by gamma diversity rather than ecological assembly mechanisms [41]. Using an extensive plant dataset, it has been found that beta diversity was primarily influenced by changes in the species pool at small scales, while local assembly mechanisms played a major role at large scale [55]. Similarly, a study on soil diazotrophic beta diversity patterns in grassland ecosystems demonstrated the importance of species pools and local community assembly processes [56]. However, the significance of these mechanisms in all soil fungal communities or specific functional groups within subtropical forest ecosystems remains to be determined. Thus, it is necessary to clearly distinguish the direct effects of species pools from the indirect effects through local community assembly processes, as this will help elucidate the mechanisms underlying the geographic patterns of soil functional fungal communities. Based on this, we predict that the beta diversity of the four functional groups of fungi is dominated by different driving mechanisms (species pool or community assembly processes) at a local scale, due to the different dispersal patterns (host-associated fungi vs free-living groups) of these functional groups.

**Fig. 1:**
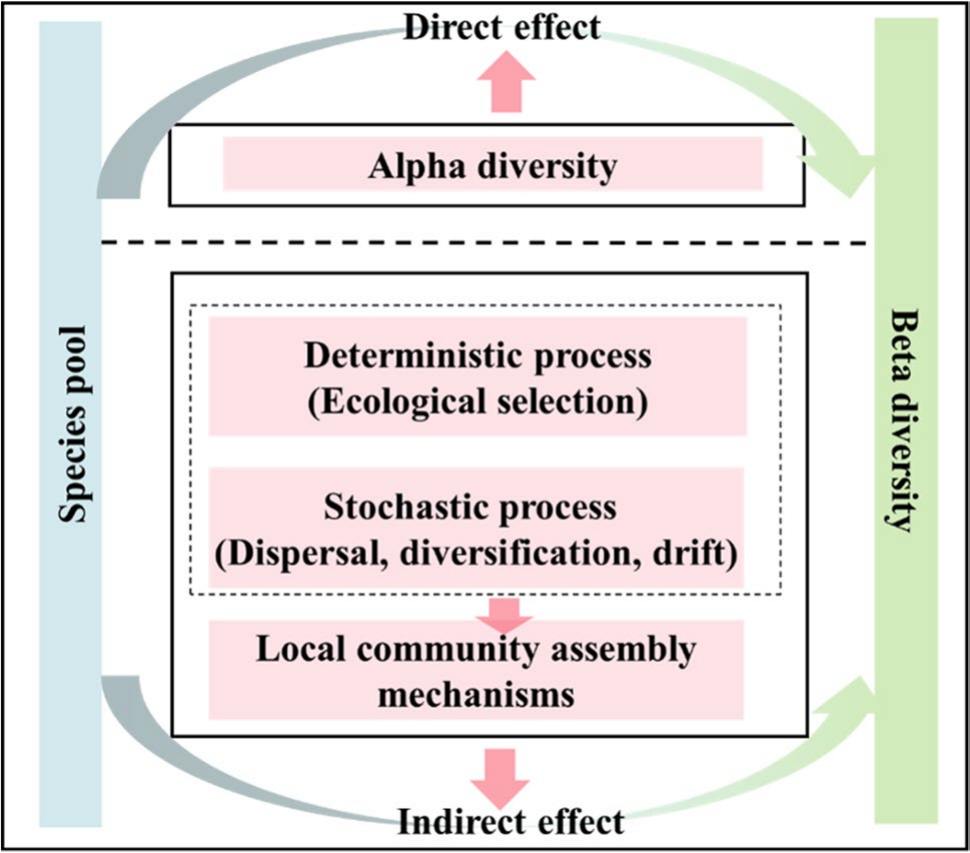
Framework illustrating the direct and indirect influences (local community assembly mechanisms) driving beta diversity through species pool dynamics

In this study, we collected 1606 soil samples from a 25-ha subtropical forest plot in Zhejiang, China. We investigated the effects of various factors (soil properties, mycorrhizal tree abundances, and topographical factors) on the four functional groups of fungi (AM, EcM, plant-pathogenic, and saprotrophic fungi). Additionally, we described the community assembly processes of these four functional groups at the same local scale. Specifically, we aimed to determine (1) whether there were differences in the diversity patterns (alpha, beta, and gamma) and community assembly mechanisms (deterministic and stochastic processes) among the four functional groups; (2) whether the beta diversity of the four functional fungal communities and the total fungi were influenced by the direct effects of species pools and the indirect effects through local ecological assembly processes (see Fig. 1); and (3) whether there were differences in the relative contributions of potential factors (soil properties, mycorrhizal tree abundances, and topographical factors) to the beta diversity of the four functional fungi.

## Materials and Methods

### Study Area

**Fig. 2:**
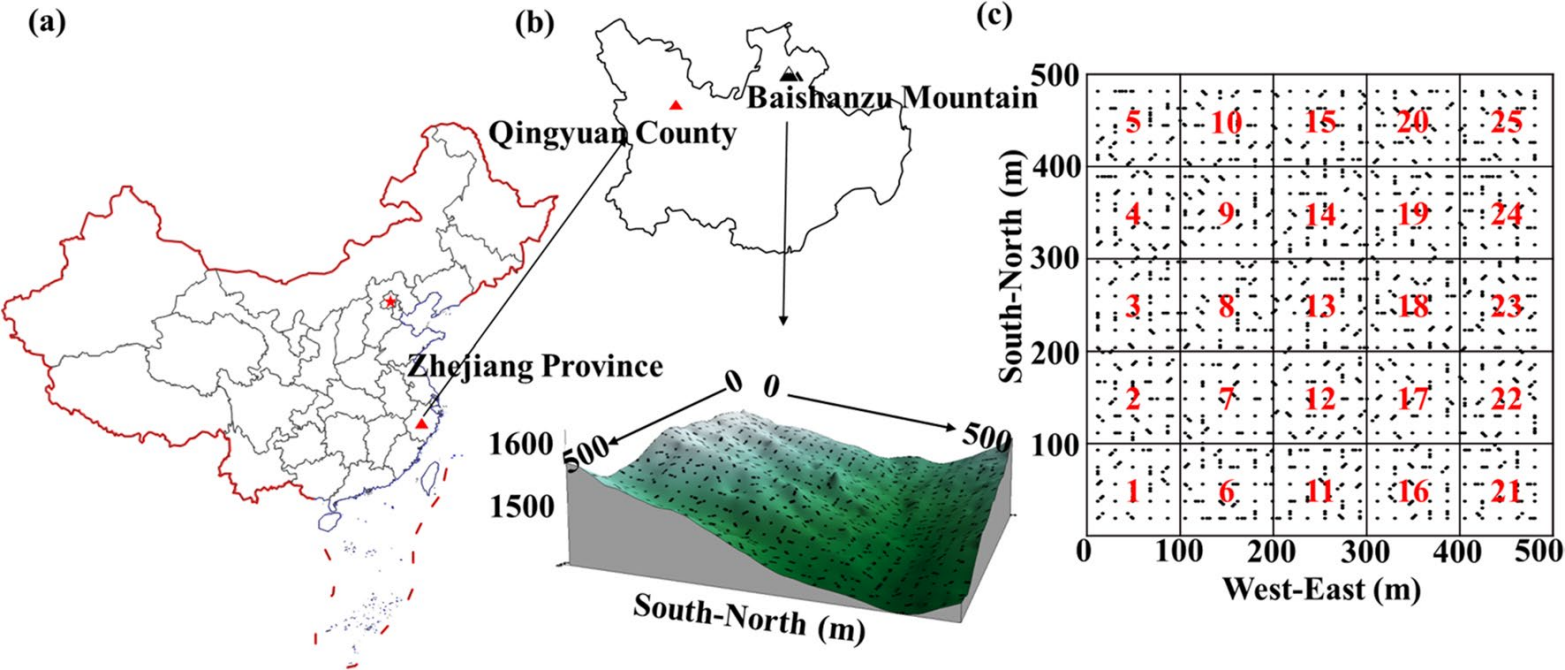
**a**–**c** Sampling distribution of the 25-ha Baishanzu (BSZ) stem-mapping forest plot. Black circles indicate the sampling points, and the names of each subplot are shown in red (1–25). The subplots were randomly distributed within a 100 m × 100 m area, resulting in a sample range of 49–78 for the 25 subplots. The red star represents Beijing, the capital of China, while the triangle represents the BSZ forest plot

The study was conducted in the Baishanzu (BSZ) Nature Reserve (27° 40′–27° 50′ N, 119° 3′–119° 6′ E), which is located in Qingyuan County, Zhejiang Province, China (Fig. 2a). The reserve primarily comprises subtropical evergreen broad-leaved tree species [57]. A 25-ha forest plot (500 m × 500 m) was established in the northern region (Fig. 2b). In 2014, all free-standing woody stems in the plot with a diameter at breast height (DBH) ≥ 1 cm were mapped, tagged, and identified to the species level. Additionally, their DBHs were measured. The study recorded a total of 204,038 stems belonging to 42 families and 86 genera (*Flora Reipublicae Popularis Sinicae*, https://www.iplant.cn/frps).

### Soil Sampling

In October 2018, soil sampling was conducted following the soil sampling protocol of the Center for Tropical Forest Sciences (CTFS) [58]. A total of 1622 soil sampling points were obtained, of which 1606 were processed (with a loss of 16 samples in the field, as shown in Fig. 2c). The sampling was conducted in two rounds, with a sampling depth of 0–10 cm and a sampling diameter of 6 cm.

To begin, the 25-ha BSZ forest plot was divided into 625 grids measuring 20 m × 20 m. Soil sampling took place at the intersections of adjacent grids, resulting in 676 grid intersections, all of which served as soil sampling points. In the second round, approximately 70% (473 out of 676) of the grid intersections were randomly selected for extended sampling. The extended sampling method followed [59]: for these selected grid intersections, one direction out of the eight possible directions (east, west, south, north, southeast, southwest, northwest, and northeast) was randomly chosen as the sampling direction. In this direction, two sampling points were randomly selected at distances of 2, 5, and 8 m. In total, 946 sampling points were obtained using this approach.

Upon collection, soil samples were immediately divided into two parts in the field. One part of the fresh soil was transported to the laboratory and stored at − 80 °C for high-throughput sequencing to obtain soil fungal data. The other part was used to measure soil physicochemical properties. Prior to analysis, this part of the soil was processed by removing small stones and fine roots and then passing it through a 2-mm sieve.

### Mycorrhizal Information for Adult Trees in the BSZ Forest Plot

Only individual adult trees with a diameter at breast height (DBH) of ≥ 10 cm were included in this study. This criterion was chosen because the symbiotic relationship between mycorrhiza and trees is influenced by the ontogenetic stage of trees, and larger-diameter trees are more likely to form mycorrhizal associations [60, 61]. Based on the available literature and information from the website http://mycorrhizas.info/index.html, tree species in the BSZ forest plot were classified into three mycorrhizal types [62]. The study included 13 ectomycorrhizal (EcM) tree species and 89 arbuscular mycorrhizal (AM) tree species (Table S1), while excluding ericoid mycorrhiza (ErM) [62]. To conduct the study, the BSZ forest plot (500 m × 500 m) was divided into 25 subplots of 100 m × 100 m each. The number of soil samples distributed within each subplot was counted, and the number of AM and EcM trees in each subplot was calculated, respectively (Fig. 2c).

### Soil Chemical Properties

To assess the role of abiotic factors in the beta diversity of the soil fungal community, we measured 16 soil variables, including soil water content, total nitrogen (TN), NH_4_^+^-N, NO_3_^−^-N, total phosphorus (TP), available phosphorus (AP), available potassium (AK), organic carbon (OC), pH, aluminum (Al), calcium (Ca), copper (Cu), iron (Fe), magnesium (Mg), manganese (Mn), and zinc (Zn) (Table S2). Soil properties were assessed using the Chinese National Standard Method (https://std.samr.gov.cn/), as well as referring to a relevant book [63]. Soil water content was determined using the aluminum box drying method. TN in the soil was measured using the Kjeldahl method. For this, fresh soil samples (10 g) were mixed with 50 mL of 2 mol/L KCl solution in a bottle, shaken for 1 h, and allowed to stand for 10 min. The supernatant was collected, and the concentrations of NH_4_^+^-N and NO_3_^−^-N were determined using a SmartChem 2000 discrete chemical analyzer. Soil TP was measured using the acid melt-molybdenum stibium anti-color method. Soil AP was determined using the sodium hydrogen carbonate (NaHCO_3_) solution-Mo-Sb anti-spectrophotometric method. We extracted the soil samples with a neutral solution of 1 mol/L^−1^ NH_4_AC, wherein NH_4_^+^-N was exchanged with K^+^ on the soil colloid surface, along with water-soluble K^+^ in the solution. The AK content in the leaching solution was determined using a flame photometer. OC was quantified using the potassium dichromate oxidation external heating method. We weighed 0.6 g of soil sample, added 6 g of a mixed flux of lithium borate and lithium carbonate, stirred and mixed thoroughly, and melted the mixture in a furnace at 1100 °C. The resulting liquid sample was poured into a mold and cooled to form glass sheets. Finally, the Al, Ca, Fe, and Mg contents were measured using an X-ray fluorescence spectrometer, while other trace elements (Cu, Mn, and Zn) in the soil samples were measured using an inductively coupled plasma atomic emission spectrometer (ICP-AES).

### Molecular Analysis of Soil Microorganisms

DNA was extracted from each soil sample using the MagPure Soil DNA KF Kit (Magigene Biotechnology Co., Ltd., Guangzhou, China). Subsequently, the DNA concentration and purity were measured using the NanoDrop One instrument (Thermo Fisher Scientific, MA, USA). For fungi, a nested polymerase chain reaction (PCR) approach was employed to target the internal transcribed spacer (ITS) region of fungal rDNA. The second ITS (ITS2) region of the rRNA operon was sequenced using the ITS3 (5′-GCATCGATGAAGAACGCAGC-3′) and ITS4 (5′-TCCTCCGCTTA TTGATATGC-3′) primer pairs [64].

Each PCR sample consisted of 25 µL of 2 × Premix Taq (Takara Biotechnology, Dalian Co. Ltd., China), 1 µL of each primer (10 mM), 20 µL of nuclease-free water, and 3 µL of DNA (20 ng/µL) template, making up a total volume of 50 µL. The PCR temperature profile included an initial cycle of 5 min at 94 °C for initialization, followed by 30 cycles of 30 s denaturation at 94 °C, 30 s annealing at 52 °C, and 30 s extension at 72 °C. This was followed by a final elongation step of 10 min at 72 °C. The length and concentration of the PCR products were determined through 1% agarose gel electrophoresis. The final mixture of PCR products was purified using the E.Z.N.A. Gel Extraction Kit (Omega, USA). The Illumina Hiseq2500 platform was utilized for PE250 sequencing of the amplicon library (Guangdong Magigene Biotechnology Co., Ltd., Guangzhou, China).

### Bioinformatics

Sequence data (.fastq) were processed using mothur software (V1.35.1) [65]. Barcodes and primers were subsequently removed, and clean tags were obtained. Sequence analyses were conducted using USEARCH software (V10) [66]. Sequences were clustered into operational taxonomic units (OTUs) with a 97% sequence similarity threshold [67]. Taxonomic information for each representative sequence was annotated using the UNITE database (for ITS) [68] with a default confidence threshold of ≥ 0.5. Fungal OTUs were classified based on ecological guilds using the FUNGuild database (http://www.stbates.org/guilds/app.php) [5]. Using the FUNGuild database, fungal OTUs in the BSZ forest plots were successfully assigned to a trophic guild with a confidence level of “probable” or “highly probable” (Table S3). Here, unmatched fungi accounted for 59.5% of total abundance in the functional groups of fungi. Prior to normalization, the original sequence numbers of the 1606 soil samples collected from the BSZ forest plot ranged from 28,843 to 367,435. Finally, in order to correct the differences in sequencing depth between samples [69, 70], the sequence of all samples was rarefied according to the minimum sequence length, and the soil samples were refined to 28,843 sequences. Here, only for the fungal dataset from the BSZ forest plot, OTUs with sequence lengths < 20 across all samples were deleted from all samples, resulting in 15,444 remaining OTUs [71].

### Biodiversity Estimation

Based on the collected fungal data, we conducted several diversity estimations for the total fungi and four functional fungal communities (i.e., AM, EcM, saprotrophic, and plant-pathogenic fungi). Firstly, we estimated the alpha diversity of each soil sample within the entire plot. Additionally, we calculated the gamma diversity for each subplot. Alpha diversity was determined by calculating the OTU richness within a single soil sample. This calculation was performed for a total of 1606 soil samples. On the other hand, gamma diversity was calculated by the total OTU richness of all soil samples within each subplot, representing a 100 m × 100 m region. To explore the direct and indirect effects of species pools on beta diversity, we employed specific methods. Beta diversity was assessed using the following formula: $\beta =1-\overline{\alpha }/\gamma$, where $\overline{\alpha }$ represents the average value of the alpha diversity across all samples within a given subplot and $\gamma$ represents the gamma diversity of that subplot [56]. It is important to note that there exist multiple methods for calculating beta diversity, and we opted for this particular approach to conduct our analysis.

### Statistical Analysis

The assembly processes of four functional groups of fungi were assessed using the normalized stochasticity ratio (*NST*) calculated with the “tNST()” function in the “NST” package [72]. First, the Bray–Curtis index was calculated for each of the four functional groups (AM, EcM, plant-pathogenic, and saprotrophic fungi) using the abundance data. This index was then compared with the corresponding Bray–Curtis index obtained from the null model. The null model was generated by randomizing the observed community 999 times, with the observed abundances randomly shifted across all the OTUs of the site × OTU table (note that this randomization does not preserve the row sum or column sum). The null model was calculated using the PF algorithm (proportional taxa occurrence frequency, fixed richness).

Next, the *NST* was calculated as follows: Let ${A}_{ij}$ be the observed similarity between two sites *i* and *j*, and ${B}_{ij}=1-{A}_{ij}$ be the observed dissimilarity. ${E}_{ij}$ is the null expected similarity between sites *i* and *j*, and ${\overline{E} }_{ij}$ means the average of 999 times. If fungal communities across sites are affected by deterministic processes and the communities become more similar, the average selection strength of communities (*SS*) is: $SS=\frac{\left(A_{ij}-\overline{E}_{ij}\right)}{A_{ij}}$. If fungal communities across sites are affected by deterministic processes and the communities become more dissimilar, the average selection strength of communities (*SS*) is: $SS=\frac{\left(B_{ij}-{\overline E}_{ij}\right)}{B_{ij}}=\frac{\left({\overline E}_{ij}-A_{ij}\right)}{\left(1-A_{ij}\right)}$. The stochasticity ratio is then defined as *ST* = 1 − *SS*. Since ${\overline{E} }_{ij}$ is not always between 0 and 1, based on this, the normalized selection strength (*NSS*) and the normalized stochasticity ratio (*NST*) are proposed to keep the final value between 0 and 1. The *NSS* is: $NSS=\frac{\left(SS-SS^T\right)}{\left(SS^D-SS^T\right)}$ Where ${SS}^{D}$ is the theoretical maximum value of *SS* under completely deterministic assembly and ${SS}^{T}$ is the theoretical minimum value of *SS* under completely stochastic processes, respectively. The *NST* is: $NST=1-NSS$. The *NST* was used to quantify the relative importance of deterministic assembly (*NST* < 50%) and stochastic processes (*NST* > 50%) for four functional groups of fungi following Ning et al. [72], respectively.

To further explore the community assembly processes of the four functional groups of fungi, we calculated the Levin’s niche breadth (*B*) index using the “niche width” function in the “spaa” package [73, 74]. The community-level *B* value (*Bcom*) represents the average *B* value in a community [75]. In this study, *Bcom* was calculated as the average of all OTUs’ niche widths for the four functional groups of fungi, corresponding to the 1606 samples.

We conducted Mantel and partial Mantel tests to assess the direct and indirect effects of species pools (gamma diversity values) and community assembly processes (*NST* values) on the beta diversity of four functional groups of fungi in the BSZ forest plot. For this analysis, we utilized the “mantel.partial” and “mantel” functions from the “vegan” package in R [76].

To identify the driving factors of beta diversity in the four functional groups, we employed variance partitioning analysis (VPA). We initially performed multicollinearity analysis using the “varclus()” function from the “Hmisc” package [77] to assess potential factors, namely soil properties, mycorrhizal tree abundances (AM and EcM tree numbers), and topographical factors (slope, elevation, and convexity). Here, we only used the Kriging interpolation method to obtain the topographic data of 1606 samples in this 25-ha plot, and total fungal data, soil property data, and mycorrhizal tree species abundance data were real measurements. All explanatory variables were standardized to equalize variances using the “scale” function in R. Model selection was performed using the stepwise method based on the Akaike information criterion (AIC) in R with the “step()” function [78, 79]. Additionally, a backward selection model, starting with the full model, was used to avoid selecting a local AIC minimum. The remaining factors identified were NH_4_^+^-N, NO_3_^−^-N, TP, AK, Al, Ca, Cu, Fe, Mg, Mn, soil water content, convexity, slope, and AM and EcM tree numbers. Data visualization was conducted using the “ggplot2” package [80], and all statistical analyses were performed in R within the RStudio environment (V4.1.1; https://www.r-project.org/).

## Results

In the total fungal community, Basidiomycota (45.6% of the total abundance) and Ascomycota (30.8% of the total abundance) exhibited the highest average relative abundances at the phylum level (Fig. S1a). Among the functional groups of fungi, EcM fungi accounted for 21.2% of the total abundance, while saprotrophic fungi represented 9.22% of the total abundance. Plant-pathogenic and AM fungi constituted 0.58% and 0.49% of the total abundance, respectively (Fig. S1b). The contour map depicted clear variations in the distribution patterns of functional groups of fungi across different sampling points (Fig. S2).

### Diversity Patterns and Community Assembly Processes of Soil Functional Fungal Communities

**Fig. 3:**
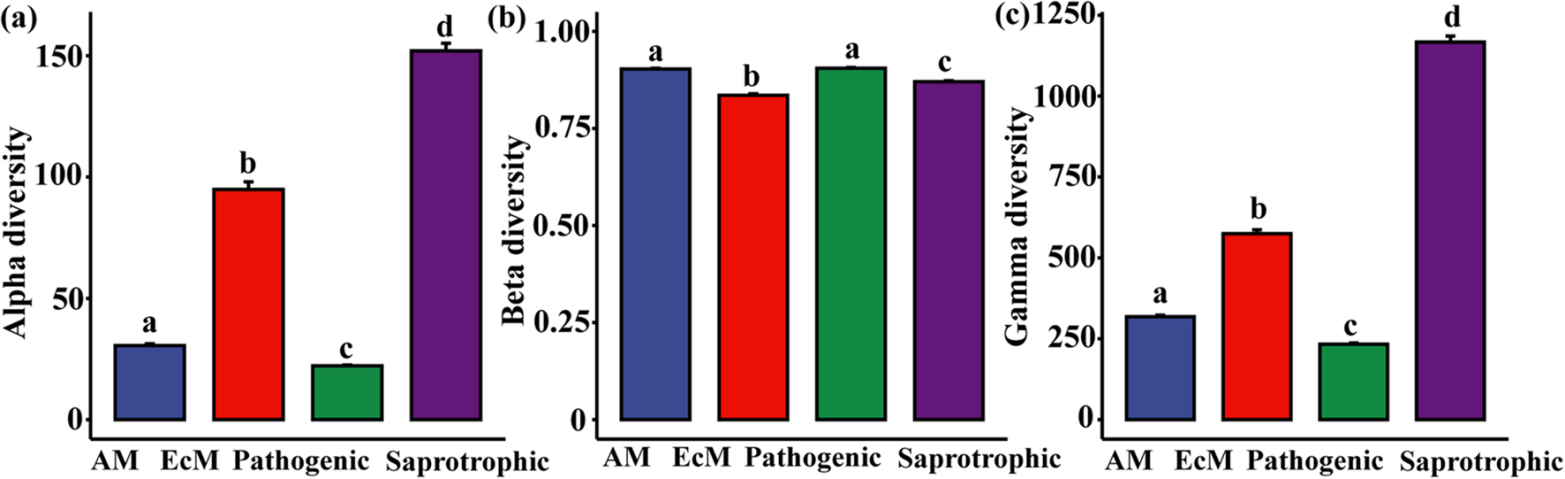
Distribution of average alpha (**a**), beta (**b**), and gamma (**c**) diversities of functional groups of soil fungi in the Baishanzu (BSZ) forest plot. The colors in green, blue, red, and purple represent plant-pathogenic (Pathogenic), arbuscular mycorrhizal (AM), ectomycorrhizal (EcM), and saprotrophic fungi, respectively. The Tukey HSD test was used for post hoc test. The different letters above bars (a, b, c, d) indicate statistically significant difference

The alpha, beta, and gamma diversities differed significantly among the fungal groups of AM, EcM, plant-pathogenic, and saprotrophic fungi (*P* < 0.001; Fig. 3a, b, c). The average alpha and gamma diversities of EcM and saprotrophic fungi exceeded that of AM and plant-pathogenic fungi throughout the entire plot (Fig. 3a, c). On the contrary, the average beta diversity of AM and plant-pathogenic fungi was higher than that of EcM and saprotrophic fungi (Fig. 3b). Additionally, the alpha, beta, and gamma diversities of the four functional fungal groups exhibited variation even within the same subplots (Fig. S3). Patterns of diversity showed limited correlation among functional groups, with only the gamma diversity of plant-pathogenic fungi displaying a positive correlation with that of the other three functional groups, while all other pairwise comparisons were nonsignificant (Fig. S4).

**Fig. 4:**
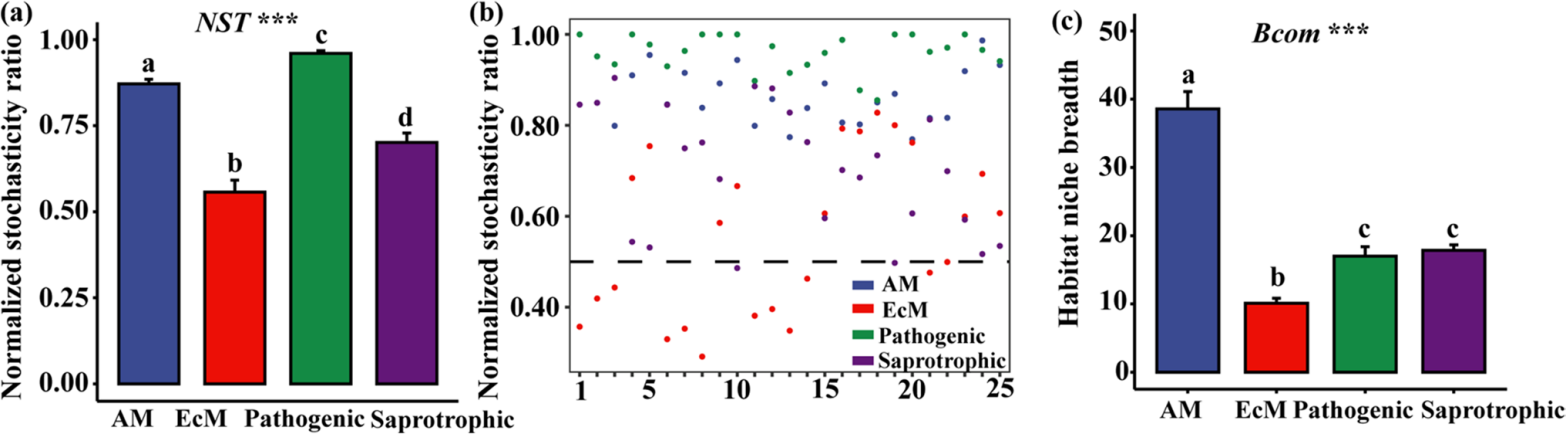
Average normalized stochasticity ratios (*NST*) of the functional fungal community (**a**). The scatter plot represents the average *NST* values of the functional fungal community across 25 subplots in the forest plot (**b**). The average niche width values (*Bcom*) are shown for four fungal groups: arbuscular mycorrhizal fungi (AM fungi), ectomycorrhizal fungi (EcM fungi), plant-pathogenic fungi (Pathogenic fungi), and saprotrophic fungi (**c**). ***P < 0.001. The *X*-axis represents the names of the 25 subplots. The Tukey HSD test was used for post hoc test. The different letters above bars (a, b, c, d) indicate statistically significant difference

The *NST* and *Bcom* values differed significantly among the fungal groups of AM, EcM, plant-pathogenic, and saprotrophic fungi (*P* < 0.001; Fig. 4a, c). Based on the *NST* values, all four groups exhibited evidence supporting the impact of stochastic processes on assembly, with this effect being particularly strong for AM and plant-pathogenic fungi (*NST* > 50%; Fig. 4a, b). In contrast, EcM and saprotrophic fungi showed more mixed evidence, with average *NST* values closer to 50% (Fig. 4a, b). Figure 4c illustrates that the mean *Bcom* value of AM fungi was the highest, indicating that AM fungi generally have a broader niche width compared to the other three functional groups.

### Species Pool Size Influences the Beta Diversity of Soil Functional Fungal Communities Directly and Indirectly Through Ecological Assembly Processes

**Fig. 5:**
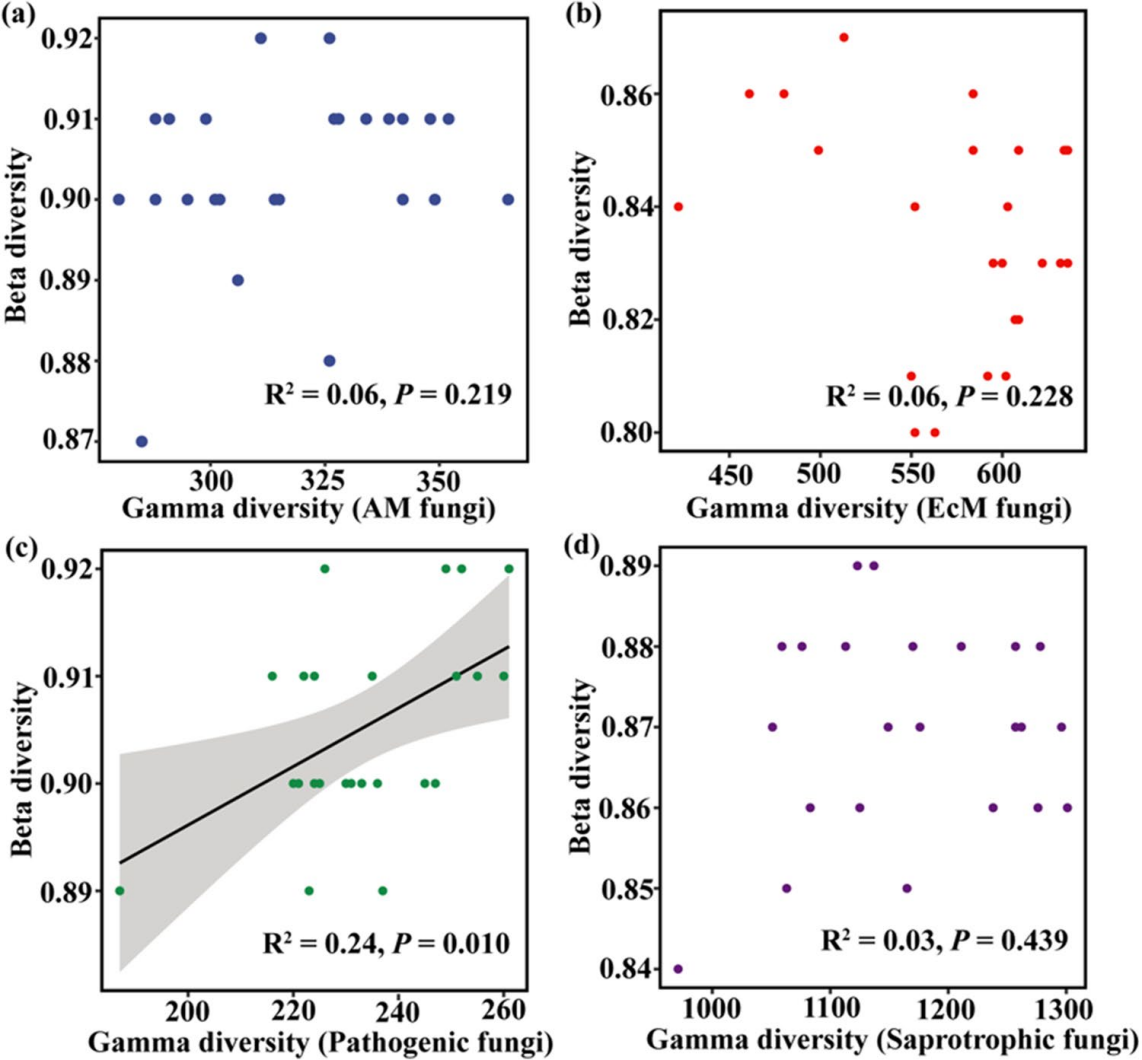
Relationships between beta diversity and gamma diversity (species pool) of different fungal functional groups. **a** Arbuscular mycorrhizal fungi (AM fungi), **b** ectomycorrhizal fungi (EcM fungi), **c** plant-pathogenic fungi, and **d** saprotrophic fungi. Solid circles in blue, red, green, and purple represent AM fungi, EcM fungi, plant-pathogenic fungi, and saprotrophic fungi, respectively. The gray areas indicate the 95% confidence intervals. Significant differences are denoted by *P* < 0.05

The size of the species pool had a direct effect only on the beta diversity of plant-pathogenic fungi and not on the other three functional groups. In the case of plant-pathogenic fungi, a significant positive correlation was observed between beta diversity and the species pool size (gamma diversity; *R*^2^ = 0.24, *P* = 0.010, Fig. 5c). However, no significant relationships were found between beta diversity and gamma diversity for AM, EcM, and saprotrophic fungi (AM fungi: *R*^2^ = 0.06, *P* = 0.219; EcM fungi: *R*^2^ = 0.06, *P* = 0.228; saprotrophic fungi: *R*^2^ = 0.03, *P* = 0.439).

We employed Mantel and partial Mantel tests to further investigate the differing direct and indirect effects (via community assembly processes) of species pools on the beta diversity of each functional fungal group. Our results demonstrated that the beta diversity of EcM fungal communities was primarily influenced indirectly through community assembly processes, rather than directly by the species pools (Mantel test: *r* = 0.48, *P* = 0.001; partial Mantel test: *r* = 0.44, *P* = 0.002; see Table 1). Conversely, the beta diversity of plant-pathogenic fungal communities was directly governed by the species pools rather than being indirectly influenced by community assembly processes (Mantel test: *r* = 0.23, *P* = 0.022; partial Mantel test: *r* = 0.18, *P* = 0.024; see Table 1). Moreover, the beta diversity of the total fungal communities in the BSZ forest plot was influenced both directly and indirectly by the species pools (Mantel test: *r* = 0.54, *P* = 0.002; *r* = 0.48, *P* = 0.001; partial Mantel test: *r* = 0.32, *P* = 0.009; *r* = 0.21, *P* = 0.014; see Table 1). In contrast, the beta diversity of AM fungal communities was not directly affected by the species pools or indirectly influenced by community assembly processes (Mantel test: *r* =  − 0.01, *P* > 0.05; *r* =  − 0.03, *P* > 0.05; partial Mantel test: *r* =  − 0.01, *P* > 0.05; *r* =  − 0.04, *P* > 0.05; see Table 1). The beta diversity of saprotrophic fungal communities also remained unaffected by both the species pools and community assembly processes (Mantel test: *r* = 0.11, *P* > 0.05; *r* = 0.12, *P* > 0.05; partial Mantel test: *r* = 0.09, *P* > 0.05; *r* = 0.02, *P* > 0.05; see Table 1).

**Table 1:** Effects of species pools and ecological assembly processes on the beta diversity of soil functional fungal communities analyzed using the Mantel test and partial Mantel test

	Mantel test	Partial Mantel test	Mantel test	Partial Mantel test
	Community assembly processes (*r*)	Controlling for species pools (*r*)	Species pools (*r*)	Controlling for community assembly processes (*r*)
AM fungi	− 0.01	0.01	− 0.03	− 0.04
EcM fungi	0.48***	0.44***	0.05	− 0.03
Plant-pathogenic fungi	− 0.05	− 0.08	0.23*	0.18*
Saprotrophic fungi	0.11	0.09	0.12	0.02
All fungi	0.54**	0.32**	0.48***	0.21*

Values indicated in bold represent significant differences (*P < 0.05, **P < 0.01, ***P < 0.001). Significant differences were determined based on 999 permutations

### The Relative Contributions of Soil Properties, Mycorrhizal Tree Abundances, and Topographical Factors to the Beta Diversity of Soil Functional Groups of Fungi

**Fig. 6:**
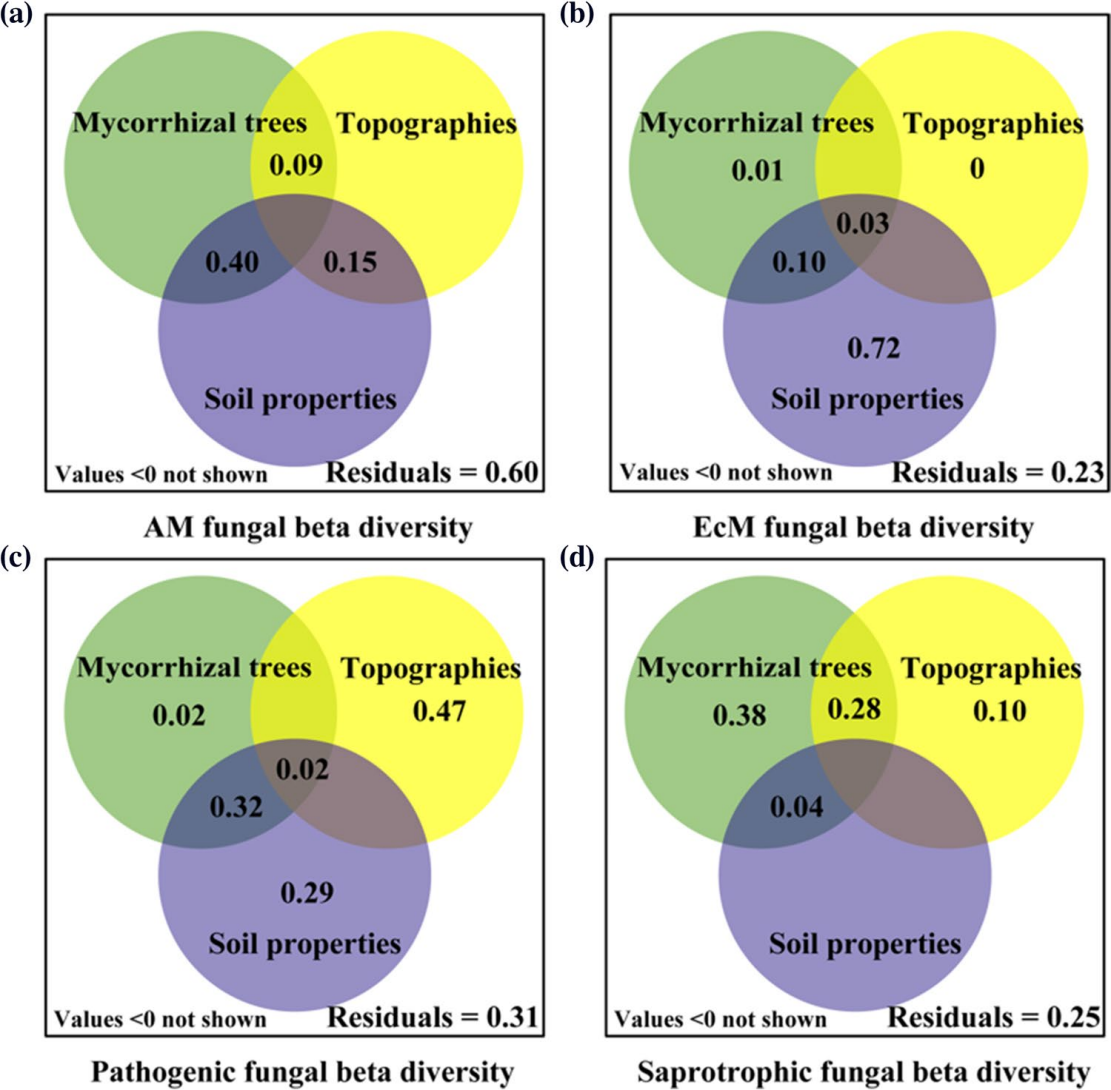
Variation partitioning analysis illustrating the proportion of variance in the beta diversity of soil functional fungi explained by soil properties, mycorrhizal tree numbers, and topographical factors. The percentages indicate the contributions, while values < 0 are not displayed. **a**, **b**, **c**, and **d** represent the beta diversity of arbuscular mycorrhizal fungi, ectomycorrhizal fungi, plant-pathogenic fungi, and saprotrophic fungi, respectively

The results of the variance partitioning analysis revealed that soil properties, mycorrhizal tree abundances, and topographical factors did not have independent effects on the beta diversity of AM fungal community. In combination, these factors only explained 40% of the total variance (Fig. 6a). However, for the other three groups, 69–77% of the variance was explained by the three sets of factors, although their relative weightings varied among the groups. Consequently, there is no single “controlling factor” for soil fungal beta diversity in this system. Specifically, soil properties were the driving factor for the beta diversity of EcM fungal community, while they had no effect on saprotrophic fungi (Fig. 6b). The beta diversity of plant-pathogenic fungi was influenced by a relative balance of soil properties and topographical factors, whereas mycorrhizal tree abundances impacted only the beta diversity of the saprotrophic fungal community (Fig. 6c, d). In addition, we examined the effects of soil properties, mycorrhizal tree abundances, and topographical factors on alpha and gamma diversity of four functional fungal communities using the variance partitioning analysis. Among them, soil properties, as the most important potential factors, had a significant impact on the alpha and gamma diversities of four functional fungal groups (Fig. S5 and Fig. S6).

## Discussion

In order to enhance our understanding of the mechanisms driving soil functional fungal communities, we conducted an evaluation of the direct and indirect effects (mediated through ecological community assembly processes) of species pools on the beta diversity of soil functional groups. This analysis was based on a dataset obtained from a 25-ha subtropical forest plot. Our study revealed that stochastic processes predominantly influenced the community assembly of AM, plant-pathogenic, and saprotrophic fungi. However, there were differences in the community assembly mechanisms of EcM fungi in different subplots. Furthermore, we found that soil properties had the most significant contribution to the beta diversity of EcM fungi, followed by plant-pathogenic fungi, while they did not independently contribute to the beta diversity of AM and saprotrophic fungi. The findings of this study hold considerable significance for advancing our comprehension of the diverse functions of fungi in forest ecosystems. By implementing a functional division of fungi based on ecological guilds [5], future research can greatly facilitate investigations into the impact of soil fungal communities on the diversity and functioning of forest ecosystems.

### Diversity Patterns of Soil Functional Fungal Communities

The biodiversity indices (alpha, beta, and gamma diversities) of the AM, EcM, plant-pathogenic, and saprotrophic fungal communities within the same subplot exhibited variation, highlighting the specificity of different functional groups (Fig. 3). Additionally, it was observed that the alpha and gamma diversities of plant-pathogenic fungi and AM fungi were similar and significantly lower than that of EcM fungi. This result can be attributed to several reasons. Firstly, sites with high rates of EcM fungi accumulation may display low rates of saprotrophic or pathogenic fungi accumulation, while sites with high rates of AM fungi accumulation tend to have high rates of plant-pathogenic fungi accumulation [81]. Based on our results, the richness distribution of four functional fungal groups was significantly observed (Fig. S2). Generally, the sites with less EcM fungi richness had more saprotrophic fungi richness, while the sites with more AM fungi richness had relatively more saprotrophic and plant-pathogenic fungi richness. Secondly, it has been suggested that EcM fungi are more resistant to pathogenic fungi compared to AM fungi, which may be linked to the fine root morphology of the host species [82, 83]. EcM fungi are also known to provide protection to plants (including seedlings) against pathogenic fungi [84–86]. In this study, EcM fungi exhibited higher diversity and stronger resistance to plant-pathogenic fungi compared to AM fungi, resulting in lower diversity of plant-pathogenic fungi in comparison. However, the findings for saprotrophic fungi differed significantly. For instance, Eagar et al. revealed that in four temperate hardwood forests in southern Indiana, United States, the diversity of plant-pathogenic and saprotrophic fungi increased with increasing dominance of AM trees, but not with EcM trees dominance [87]. In this study, we found that EcM tree species were dominant by comparing the relative basal areas of the two mycorrhizal species (Fig. S7). At the same time, we found that the relative basal area of EcM tree species was significantly negatively correlated with the diversity of AM fungi. The diversity of EcM fungi increased with the increase of EcM tree species dominance, while the diversity of plant-pathogenic and saprotrophic fungi decreased with the increase of EcM tree species dominance, but none of them was significant (Fig. S8). This result was consistent with previous study [87].

### Community Assembly Processes of Soil Functional Fungal Communities

In this study, the community assembly processes of EcM fungi were predominantly determined by deterministic processes in 12 subplots (BSZ1, BSZ2, BSZ3, BSZ6, BSZ7, BSZ8, BSZ11, BSZ12, BSZ13, BSZ14, BSZ21, and BSZ22) and stochastic processes in the remaining 13 subplots (Fig. 4). This distinction may be attributed to the influence of elevation on the distribution of EcM fungi in the 25 subplots (Fig. S9) [88]. In this study, there was a significant correlation between EcM fungal diversity and elevation factor (*R*^2^ = 0.38, *P* < 0.001). A recent study conducted in mountains of southwest China also demonstrated that elevation has a significant impact on EcM fungal communities [89]. It was further inferred that the differences in community assembly processes of EcM fungi in different subplots might be caused by the elevation. Moreover, EcM fungal communities at different elevations within the BSZ plot could be influenced by various factors, such as soil water content and the distribution of aboveground mycorrhizal tree species. These factors may also result in different community assembly mechanisms [30, 90].

Based on the *NST* and *Bcom* values, this study revealed that EcM fungi exhibited the narrowest average niche width and the least spatially even distribution compared to the other three functional fungal groups (Fig. S2). One possible explanation for this pattern is that species with smaller niche widths tend to have more uneven distributions and are more susceptible to dispersal limitation. Conversely, species with larger niche widths tend to be more widely and evenly distributed and less affected by dispersal limitation [91, 92]. Compared with other three functional fungal groups, EcM fungi had the smallest average *NST* value. The smaller *NST* value means that it is more susceptible to deterministic processes (environmental factors) [72]. EcM fungi are more susceptible to environmental changes when it involves global change or local disturbances.

### Driving Mechanism Underlying Beta Diversity of Soil Functional Fungi

Our findings revealed a significant positive correlation between the beta diversity and gamma diversity of plant-pathogenic fungi within functional fungal communities. This result aligns with previous studies conducted in other ecosystems [41, 56, 93, 94]. For instance, Grman et al. demonstrated that the beta diversity of plant communities in grassland ecosystems increases with the species pool [93], while Wang et al. reported that an increase in gamma diversity leads to an increase in the beta diversity of diazotrophic communities [56]. However, we did not observe a significant relationship between beta diversity and gamma diversity in the other three functional fungal groups investigated in this study (AM, EcM, and saprotrophic fungi). Generally, according to the framework, the gamma diversity (i.e., species pool) can influence the beta diversity of fungal communities either directly or indirectly (community assembly processes). Here, we did not find a direct correlation between gamma diversity and beta diversity in the other three functional fungi groups, which may be due to the indirect driving role of community assembly processes. The processes of community assembly are closely related to biological type, environmental filtration, and biological interactions [95–97].

Our results partially support the hypothesis that the beta diversity of microbial communities is influenced both directly and indirectly by species pools and community assembly processes, addressing our second research question. Specifically, the beta diversity of the EcM fungal community was primarily driven indirectly through community assembly processes rather than by species pools (Table 1). The influence of community assembly processes suggests that different selective filters, such as homogeneous environments, heterogeneous environments, and dispersal processes, can shape beta diversity by selectively filtering species from the species pool in distinct ways [98, 99]. For example, Pereira et al. studied the effects of two tropical rain forest types (semi-deciduous rain forest and dense ombrophilous forests) on AM fungal community assembly processes in the Atlantic rain forest in Northeastern Brazil, South America. The results showed that there were significant differences in community assembly dynamics between the two forest types, and the differences caused by the host tree presence or preferences are most likely due to different responses to environmental variables [51].

It is increasingly recognized that soil functional fungal communities may be governed by different factors, and different functional groups within the same community may exhibit similar or distinct drivers [32, 33]. In our study, we also identified soil properties, mycorrhizal tree numbers, and topographic factors as the most significant factors influencing the beta diversity of EcM, saprotrophic, and plant-pathogenic fungi, respectively. The beta diversity of AM fungi, on the other hand, was influenced by a combination of soil properties and mycorrhizal tree species. And the three potential factors had no independent effects on the beta diversity of AM fungal community. Furthermore, based on the Venn diagrams, we observed that the residuals (unexplained by these factors) of the AM fungal community were the largest (60%), suggesting a potential influence of unmeasured environmental variables and ecological processes. It might also be due to the strong effect of stochastic processes. Additionally, it is important to note that since our study captured a snapshot in time, different results could emerge due to temporal and seasonal changes.

## Conclusions

This study investigates the impact of species pools, both direct and indirect (through community assembly processes), on the beta diversity of various functional fungal groups within a 25-ha forest plot in northern China. The findings emphasize the importance of classifying fungal communities into distinct functional groups for research purposes. Additionally, by analyzing the potential driving mechanisms behind four functional fungi, we gain a more comprehensive understanding of the significant variations in driving factors among different functional groups within specific ecosystems.

### Supplementary Information

Below is the link to the electronic supplementary material.

Supplementary file1 (DOCX 3959 KB)

## Data Availability

The datasets associated with this study, including the complete OTU sequences, are available on figshare at https://figshare.com/articles/dataset/Soil_fungal_community_data_of_Baishanzu_plot/25512139/1.
