# Supplementary material for: Local Community Assembly Mechanisms and the Size of Species Pool Jointly Explain the Beta Diversity of Soil Fungi
Source: Microb Ecol. 2024 Apr 11;87:58. doi: 10.1007/s00248-024-02374-3 (PMC11008070; doi:10.1007/s00248-024-02374-3)
Supplement: Supplementary file 1 [file 248_2024_2374_MOESM1_ESM.docx]

**Supplementary material**

**Local community assembly mechanisms and the size of species pool jointly explain the beta diversity of soil fungi**

Hua Xing^1,2^, Wuwei Chen^3^, Yu Liu^1,4^*, James F. Cahill Jr^2^

^1^ECNU-Alberta Joint Lab for Biodiversity Study, Tiantong Forest Ecosystem National Observation and Research Station, School of Ecological and Environmental Sciences, East China Normal University, Shanghai 200241, China.

^2^Department of Biological Sciences, University of Alberta, Edmonton, AB T6G 2E9, Canada.

^3^Qingyuan Bureau Natural Resources and Planning, Qingyuan, 323800, China.

^4^Shanghai Institute of Pollution Control and Ecological Security, Shanghai 200082, China.

*Correspondence author. School of Ecological and Environmental Sciences, East China Normal University, 500 Dongchuan Road, Minhuang District, Shanghai 200241, China. E-mail address: yuliu@des.ecnu.edu.cn (Y. Liu).

**Figure Legends**

**Fig. S1** Composition of fungal taxa at the level of phylum (a) and the relative abundances of soil functional fungi (b) from 25 subplots in the Baishanzu forest plot. Functional guilds were identified based on the FUNGuild database. Note: Unmatched functional fungal taxa were not included.

**Fig. S2** The richness distribution of operational taxonomic unit (OTU) of soil functional fungi at the 50 m scale. Functional fungi are as follows: arbuscular mycorrhizal (AM) fungi (a), ectomycorrhizal (EcM) fungi (b), plant-pathogenic fungi (c) and saprotrophic fungi (d).
**Fig. S3** Distribution of alpha (a), beta (b), and gamma (c) diversities of functional groups of soil fungi across 25 subplots in the Baishanzu forest plot. The points in green, blue, red, and purple represent plant-pathogenic (Pathogenic), Arbuscular mycorrhizal (AM), Ectomycorrhizal (EcM), and Saprotrophic fungi, respectively. The X-axis displays the names of the 25 subplots.

**Fig. S4** The linear relationship between alpha, beta and gamma diversity of three functional fungi and that of plant-pathogenic fungi. Functional fungi are as follows: arbuscular mycorrhizal (AM) fungi (a, d, g), ectomycorrhizal (EcM) fungi (b, e, h), and saprotrophic fungi (c, f, i).

**Fig. S5** Variation partitioning analysis illustrating the proportion of variance in the alpha diversity of soil functional fungi explained by soil properties, mycorrhizal tree numbers, and topographical factors. The percentages indicate the contributions, while values < 0 are not displayed. Panels (a), (b), (c), and (d) represent the alpha diversity of arbuscular mycorrhizal fungi, ectomycorrhizal fungi, plant-pathogenic fungi, and saprotrophic fungi, respectively.

**Fig. S6** Variation partitioning analysis illustrating the proportion of variance in the gamma diversity of soil functional fungi explained by soil properties, mycorrhizal tree numbers, and topographical factors. The percentages indicate the contributions, while values < 0 are not displayed. Panels (a), (b), (c), and (d) represent the gamma diversity of arbuscular mycorrhizal fungi, ectomycorrhizal fungi, plant-pathogenic fungi, and saprotrophic fungi, respectively.

**Fig. S7** Box plot of the relative basal area of arbuscular mycorrhizal (AM) trees and ectomycorrhizal (EcM) trees in the Banshanze plot.

**Fig. S8** The linear relationship between alpha diversity of four functional fungi and relative EcM basal area. Functional fungi are as follows: arbuscular mycorrhizal (AM) fungi (a), ectomycorrhizal (EcM) fungi (b), plant-pathogenic fungi (c), and saprotrophic fungi (d).

**Fig. S9** Sampling distribution of the 25-ha Baishanzu stem-mapping plot. Black circles indicated sampling points. The names of 12 subplots were in green (ectomycorrhizal (EcM) fungi were dominated by deterministic processes). The names of 13 subplots were in red (EcM fungi were dominated by stochastic processes; a). The distribution of water content in the Banshanzu forest plot (b). The distribution of arbuscular mycorrhizal (AM) tree numbers (DBH ≥20 cm) in the Baishanzu forest plot (c). The distribution of EcM tree numbers (DBH ≥20 cm) in the Baishanzu forest plot (d).

**Table S1** Mycorrhizal (arbuscular mycorrhizal, AM and ectomycorrhizal, EcM) types of tree species in the Baishanzu forest plot.

**Table S2** Soil properties in the Baishanzu plot.

**Table S3** Number of operational taxonomic units (OTUs) of rhizosphere fungi in each trophic mode and functional guild for all samples in the Baishanzu plot.

**Fig. S1** Composition of fungal taxa at the level of phylum (a) and the relative abundances of soil functional fungi (b) from 25 subplots in the Baishanzu forest plot. Functional guilds were identified based on the FUNGuild database. Note: Unmatched functional fungal taxa were not included.

**Fig. S2** The richness distribution of operational taxonomic unit (OTU) of soil functional fungi at the 50 m scale. Functional fungi are as follows: arbuscular mycorrhizal (AM) fungi (a), ectomycorrhizal (EcM) fungi (b), plant-pathogenic fungi (c) and saprotrophic fungi (d).

**Fig. S3** Distribution of alpha (a), beta (b), and gamma (c) diversities of functional groups of soil fungi across 25 subplots in the Baishanzu forest plot. The points in green, blue, red, and purple represent plant-pathogenic (Pathogenic), Arbuscular mycorrhizal (AM), Ectomycorrhizal (EcM), and Saprotrophic fungi, respectively. The X-axis displays the names of the 25 subplots.

**Fig. S4** The linear relationship between alpha, beta and gamma diversity of three functional fungi and that of plant-pathogenic fungi. Functional fungi are as follows: arbuscular mycorrhizal (AM) fungi (a, d, g), ectomycorrhizal (EcM) fungi (b, e, h), and saprotrophic fungi (c, f, i).

**Fig. S5** Variation partitioning analysis illustrating the proportion of variance in the alpha diversity of soil functional fungi explained by soil properties, mycorrhizal tree numbers, and topographical factors. The percentages indicate the contributions, while values < 0 are not displayed. Panels (a), (b), (c), and (d) represent the alpha diversity of arbuscular mycorrhizal fungi, ectomycorrhizal fungi, plant-pathogenic fungi, and saprotrophic fungi, respectively.

**Fig. S6** Variation partitioning analysis illustrating the proportion of variance in the gamma diversity of soil functional fungi explained by soil properties, mycorrhizal tree numbers, and topographical factors. The percentages indicate the contributions, while values < 0 are not displayed. Panels (a), (b), (c), and (d) represent the gamma diversity of arbuscular mycorrhizal fungi, ectomycorrhizal fungi, plant-pathogenic fungi, and saprotrophic fungi, respectively.

**Fig. S7** Box plot of the relative basal area of arbuscular mycorrhizal (AM) trees and ectomycorrhizal (EcM) trees in the Baishanzu plot.

**Fig. S8** The linear relationship between alpha diversity of four functional fungi and relative EcM basal area. Functional fungi are as follows: arbuscular mycorrhizal (AM) fungi (a), ectomycorrhizal (EcM) fungi (b), plant-pathogenic fungi (c), and saprotrophic fungi (d).

**Fig. S9** Sampling distribution of the 25-ha Baishanzu stem-mapping plot. Black circles indicated sampling points. The names of 12 subplots were in green (ectomycorrhizal (EcM) fungi were dominated by deterministic processes). The names of 13 subplots were in red (EcM fungi were dominated by stochastic processes; a). The distribution of water content in the Baishanzu forest plot (b). The distribution of arbuscular mycorrhizal (AM) tree numbers (DBH ≥20 cm) in the Baishanzu forest plot (c). The distribution of EcM tree numbers (DBH ≥20 cm) in the Baishanzu forest plot (d).

.

**Table S1** Mycorrhizal (arbuscular mycorrhizal, AM and ectomycorrhizal, EcM) types of tree species in the Baishanzu forest plot.

| Tree species | Family | Mycorrhizal type | Tree species | Family | Mycorrhizal type |
| --- | --- | --- | --- | --- | --- |
| *Quercus engleriana* | Fagaceae | EcM | *Magnolia cylindrica* | Magnoliaceae | AM |
| *Symplocos paniculata* | Symplocaceae | AM | *Pinus taiwanensis* | Pinaceae | EcM |
| *Symplocos anomala* | Symplocaceae | AM | *Styrax calvescens* | Styracaceae | AM |
| *Hovenia dulcis* | Rhamnaceae | AM | *Padus grayana* | Rosaceae | AM |
| *Viburnum setigerum* | Adoxaceae | AM | *Morus australis* | Moraceae | AM |
| *Ilex crenata* | Aquifoliaceae | AM | *Illicium jiadifengpi* | Schisandraceae | AM |
| *Eurya alata* | Pentaphylacaceae | AM | *Stewartia sinensis* | Theaceae | AM |
| *Meliosma flexuosa* | Sabiaceae | AM | *Smilax arisanensis* | Smilacaceae | AM |
| *Adinandra glischroloma* | Pentaphylacaceae | AM | *Sorbus hemsleyi* | Rosaceae | AM |
| *Euonymus myrianthus* | Celastraceae | AM | *Daphniphyllum macropodium* | Daphniphyllaceae | AM |
| *Bothrocaryum controversum* | Cornaceae | AM | *Ilex pedunculosa* | Aquifoliaceae | AM |
| *Erythroxylum sinense* | Erythroxylaceae | AM | *Acer amplum* | Sapindaceae | AM |
| *Pyrus calleryana* | Rosaceae | AM | *Nyssa sinensis* | Cornaceae | AM |
| *Lithocarpus brevicaudatus* | Fagaceae | EcM | *Carpinus viminea* | Betulaceae | EcM |
| *Cyclobalanopsis multinervis* | Fagaceae | EcM | *Camellia fraterna* | Theaceae | AM |
| *Liriodendron chinense* | Magnoliaceae | AM | *Betula luminifera* | Betulaceae | EcM |
| *Fokienia hodginsii* | Cupressaceae | AM | *Padus buergeriana* | Rosaceae | AM |
| *Microtropis fokienensis* | Celastraceae | AM | *Cryptomeria fortunei* | Cupressaceae | AM |
| *Eurya muricata* | Pentaphylacaceae | AM | *Pinus massoniana* | Pinaceae | EcM |
| *Sympocos lucida* | Symplocaceae | AM | *Acer pubinerve* | Sapindaceae | AM |
| *Symplocos lancifolia* | Symplocaceae | AM | *Toxicodendron trichocarpum* | Anacardiaceae | AM |
| *Fagus lucida* | Fagaceae | EcM | *Cerasus serrulata var. pubescens* | Rosaceae | AM |
| *Viburnum sympodiale* | Adoxaceae | AM | *Ilex triflora* | Aquifoliaceae | AM |
| *Cyclobalanopsis stewardiana* | Fagaceae | EcM | *Schima superba* | Theaceae | AM |
| *Meliosma oldhamii* | Sabiaceae | AM | *Ilex litseifolia* | Aquifoliaceae | AM |
| *Lindera erythrocarpa* | Lauraceae | AM | *Osmanthus* | Oleaceae | AM |
| *Ternstroemia gymnanthera* | Pentaphylacaceae | AM | *Taxus chinensis var. mairei* | Taxaceae | AM |
| *Magnolia officinalis* | Magnoliaceae | AM | *Symplocos confusa* | Symplocaceae | AM |
| *Cleyera pachyphylla* | Pentaphylacaceae | AM | *Machilus pauhoi* | Lauraceae | AM |
| *Clethra barbinervis* | Clethraceae | AM | *Rhus potaninii* | Anacardiaceae | AM |
| *Litsea elongata* | Lauraceae | AM | *Cyclobalanopsis glauca* | Fagaceae | EcM |
| *Ilex ficoidea* | Aquifoliaceae | AM | *Acer davidii* | Sapindaceae | AM |
| *Lindera obtusiloba* | Lauraceae | AM | *Euonymus hamiltonianus* | Celastraceae | AM |
| *Idesia polycarpa* | Salicaceae | AM | *Cyclobalanopsis gracilis* | Fagaceae | EcM |
| *Cunninghamia lanceolata* | Cupressaceae | AM | *Ilex editicostata* | Aquifoliaceae | AM |
| *Rhaphiolepis indica* | Rosaceae | AM | *Corylopsis glandulifera* | Hamamelidaceae | AM |
| *Sorbus folgneri* | Rosaceae | AM | *Ilex suaveolens* | Aquifoliaceae | AM |
| *Dendropanax dentiger* | Araliaceae | AM | *Pterostyrax corymbosus* | Styracaceae | AM |
| *Sycopsis sinensis* | Hamamelidaceae | AM | *Photinia parvifolia* | Rosaceae | AM |
| *Ilex formosana* | Aquifoliaceae | AM | *Acer elegantulum* | Sapindaceae | AM |
| *Castanopsis eyrei* | Fagaceae | EcM | *Cornus hongkongensis subsp. elegans* | Cornaceae | AM |
| *Symplocos glomerata* | Symplocaceae | AM | *Pittosporum illicioides* | Pittosporaceae | AM |
| *Eurya hebeclados* | Pentaphylacaceae | AM | *Styrax japonicus* | Styracaceae | AM |
| *Ilex wilsonii* | Aquifoliaceae | AM | *Toxicodendron succedaneum* | Anacardiaceae | AM |
| *Acanthopanax evodiaefolius* | Araliaceae | AM | *Ficus heteromorpha* | Moraceae | AM |
| *Halesia macgregorii* | Styracaceae | AM | *Skimmia reevesiana* | Rutaceae | AM |
| *Lithocarpus hancei* | Fagaceae | EcM | *Cerasus campanulata* | Rosaceae | AM |
| *Hydrangea paniculata* | Hydrangeaceae | AM | *Ilex tsoi* | Aquifoliaceae | AM |
| *Eurya rubiginosa var. attenuata* | Pentaphylacaceae | AM | *Sorbus dunnii* | Rosaceae | AM |
| *Neolitsea aurata var.* | Lauraceae | AM | *Symplocos sumuntia* | Symplocaceae | AM |
| *Cerasus schneideriana* | Rosaceae | AM | *Photinia beauverdiana* | Rosaceae | AM |

**Table S2** Soil properties in the Baishanzu plot.

|  | Minimum | Maximum | Mean | Std.dev | coef.var | p |
| --- | --- | --- | --- | --- | --- | --- |
| Water content（%） | 17.710 | 91.370 | 45.453 | 9.073 | 0.200 | <0.001 |
| NH_4_^+^-N (mg/kg) | 0.370 | 184.326 | 19.970 | 14.253 | 0.714 | <0.001 |
| NO_3_^-^-N (mg/kg) | 0.042 | 62.472 | 3.719 | 5.670 | 1.525 | <0.001 |
| TN（%） | 0.105 | 2.013 | 0.566 | 0.235 | 0.414 | <0.001 |
| TP (g/kg） | 0.058 | 1.136 | 0.375 | 0.150 | 0.400 | <0.001 |
| AP (mg/kg) | 0.300 | 166.700 | 12.819 | 17.909 | 1.397 | <0.001 |
| pH | 0.360 | 5.770 | 4.251 | 0.365 | 0.086 | <0.001 |
| AK (mg/kg) | 60.326 | 315.507 | 176.695 | 43.867 | 0.248 | <0.05 |
| OC（%） | 1.291 | 50.425 | 9.617 | 4.874 | 0.507 | <0.001 |
| Al（%） | 1.039 | 11.252 | 7.276 | 1.053 | 0.145 | <0.001 |
| Ca（%） | 0.049 | 1.143 | 0.117 | 0.045 | 0.384 | <0.001 |
| Cu（%） | 1.954 | 119.048 | 7.925 | 4.350 | 0.549 | <0.001 |
| Fe（%） | 0.541 | 4.440 | 2.947 | 0.565 | 0.192 | <0.001 |
| Mg（%） | 0.120 | 1.312 | 0.244 | 0.056 | 0.229 | <0.001 |
| Mn (mg/kg) | 42.823 | 3086.552 | 336.558 | 229.882 | 0.683 | <0.001 |
| Zn (mg/kg) | 23.549 | 190.795 | 67.291 | 19.046 | 0.283 | <0.001 |

**Table S3** Number of operational taxonomic units (OTUs) of rhizosphere fungi in each trophic mode and functional guild for all samples in the Baishanzu plot.

| Trophic mode | OTUs | Functional guild | OTUs |
| --- | --- | --- | --- |
| All Fungi | 15444 |  |  |
| Saprotrophs | 2021 |  |  |
| Symbiotrophs | 1802 | Arbuscular mycorrhiza | 472 |
|  |  | Ectomycorrhiza | 1028 |
|  |  | Others | 302 |
| Pathotrophs | 569 | Plant pathogen | 414 |
|  |  | Others | 155 |
| Multiple trophic modes | 1222 |  |  |
| Unmatched | 9830 |  |  |
